# Supplementary material for: Targeted redox inhibition of protein phosphatase 1 by Nox4 regulates eIF2α‐mediated stress signaling
Source: EMBO J. 2016 Jan 7;35(3):319–34. doi: 10.15252/embj.201592394 (PMC4741303; doi:10.15252/embj.201592394)
Supplement: Supplementary file 1 — Appendix [file EMBJ-35-319-s001.pdf]

APPENDIX

Targeted redox inhibition of protein phosphatase 1 by Nox4 regulates eIF2α-mediated stress signaling

Celio X.C. Santos,[1] Anne D. Hafstad,[1,2] Matteo Beretta,[1] Min Zhang,[1] Chris Molenaar,[1] Jola Kopec,[1,3] Dina Fotinou,[1,3] Thomas V. Murray,[1] Andrew M. Cobb,[1] Daniel Martin,[1] Maira Zeh Silva,[1,3] Narayana Anilkumar,[1] Katrin Schröder,[4] Catherine M. Shanahan,[1] Alison C. Brewer,[1] Ralf P. Brandes,[4] Eric Blanc,[5] Maddy Parsons,[3] Vsevelod Belousov,[6] Richard Cammack,[7] Robert C. Hider,[7] Roberto A. Steiner,[3] Ajay M. Shah[1]

[1]Cardiovascular Division and [3]Randall Division, King's College London British Heart Foundation Centre of Excellence, London, UK;

[2]Cardiovascular Research Group, Department of Medical Biology, The Arctic University of Norway, Tromsø, Norway;

[4]Institute for Cardiovascular Physiology, Goethe-University, Frankfurt, Germany;

[5]MRC Centre for Developmental Neurobiology, King's College London, UK;

[6]Shemyakin-Ovchinnikov Institute of Bioorganic Chemistry, Moscow, Russia;

[7]Institute of Pharmaceutical Science, King's College London, UK.

MATERIALS AND METHODS

Unless otherwise indicated, all chemicals were purchased from Sigma Aldrich or Calbiochem and were of analytical or higher purity grade.

Cells. Primary cultures of neonatal rat cardiomyocytes were prepared using standard methods (Zhang et al, 2010). Rat H9c2 cardiomyoblasts, HEK293 and U2OS cells were from ATCC. MEFs were prepared from 13.5 day-old embryos of Nox4$^{-/-}$ and littermate WT mice, and immortalized with SV40 large T antigen.

Transfections. Plasmids were transfected into HEK293 or U2OS cells using Lipofectamine™ 2000 (Invitrogen) and into MEFs using FuGENE reagent (Promega) or TurboFect (Fisher) in the case of HyPer probes, according to the manufacturer's instructions. Experiments were performed 24 h after transfection. Plasmid sources were: ATF4 (Addgene #24874), GADD34-Flag Mouse MyD116.PFLAG.CMV2 (Addgene # 21834), GADD34 Mouse MyD116.delC.pBABEpu (Addgene # 21835) and pBABE-puro SV40 LT (Addgene # 13970). In some experiments, cardiac cells were transduced with adenovirus expressing Nox4 (Ad.Nox4), β-galactosidase (Ad.βGal), a short hairpin sequence targeted against Nox4 (Ad.shRNA.Nox4) or a short hairpin sequence targeted against GFP as control (Ad.Ctl) at a multiplicity of infection (MOI) of 20, and cells were used 48 h later (Peterson et al, 2009; Zhang et al, 2010). Short interfering RNAs (siRNAs) were purchased from Qiagen and were transfected using Hyperfect reagent (Qiagen). siRNA sequences were: rat Nox4 F:5`-CCAUUAUCUCAGUAAUCAATT-3`, R:5`-UUGAUUACUGAGAUAAUGGTG-3`; rat ATF4(1): F:5`GCCUAGGUCUCUUAGAUGATT-3`, R: 5`-UCAUCUAAGAGACCUAGGCTT-3`;

rat ATF4(2): F:5`GAAGAGGUCCGUAAGGAATT-3`, R: 5`-UUGCCUUACGGACCUCUUCTA-3`.

Nox4 constructs. Nox4 deletion constructs were generated from a pcDNA3.1-full length Nox4-myc plasmid (Anilkumar et al, 2008) by site-directed mutagenesis. The pcDNA3.1-Nox4-TD construct involved deletion of all amino acid residues subsequent to Met358. The pEF-Nox4-CD-Myc construct was generated by inserting a region of the cytosolic domain of pcDNA Nox4 between the NotI and NcoI sites of pEF-NLS-myc (Invitrogen). The cytosolic domain cDNA was generated with the primer pair 5'-GGAATTCCATGGCGGTGTCCTGGAG-3' (incorporating a NotI restriction site) and 5'-GGAATTGCGGCCGCGCTGAAGGAGGATTCTTTAT-3' (incorporating a NcoI restriction site) using Herculase (Stratagene) (Anilkumar et al, 2013). The NLS sequence was deleted from the plasmid. The Nox4 P437H mutant was subcloned from the pcDNA3.1-full length Nox4. The Nox4 P437H mutation inhibits NADPH binding and ROS generation in NADPH oxidases (Dinauer et al, 1989; Debeurme et al, 2010). All constructs were verified by sequencing.

Confocal microscopy. Cells were grown on 12 mm round coverslips and fixed in 3% formaldehyde/PBS pH7.4 containing 0.025% glutaraldehyde. Cells were permeabilized with 0.1% Triton for 3 min and incubated with primary antibodies: rabbit anti-Nox4 (1:250, Anilkumar et al, 2008); mouse anti-KDEL (1:500, Enzo Life Sciences); mouse anti-FLAG (1:500, Sigma Aldrich); rabbit anti-Myc (1:500, Santa Cruz); rabbit anti-GADD34 (1:500, Santa Cruz); mouse anti-GADD34 (1:500, Novus Biological); and mouse anti-PDI (1:500, Enzo Life Sciences). Secondary antibodies were goat anti-mouse Alexa 568 (1:500, ThermoFisher) and goat anti-rat Alexa 488

(1:500, ThermoFisher). Cells were mounted in Mowiol, and imaging was performed using a 100x 1.49 NA objective on a Nikon Ti-Eclipse microscope equipped with a Yokagawa CSU-X1-M2 spinning disk unit, and an Andor Neo sCMOS camera. Coherent lasers (405, 488, 561nm) were coupled to the CSU-X1 with optical fibers. The dichroic mirror in the spinning-disk unit was a Di01-T405/488/568/647 from Semrock Inc. A Sutter instruments filter wheel with Chroma emission filters was used. Typically, a Z-stack of 11 steps over 3.0 micron was acquired and the maximum projection image was used for display and comparison of expression levels. The same acquisition and image contrast settings were used for control and treated cells.

3D Structural Illumination microscopy (SIM) was performed on a NIKON SIM system equipped with a 100 x 1.49 NA PlanApo oil immersion objective, an Andor EMCCD camera, and 488 and 567 nm diode lasers. Structured illumination image stacks were acquired with a z-distance of 100 nm and with 15 raw images per plane, 5 phases, 3 angles. The structured illumination raw data were computationally reconstructed using the SIM-module in the NIS Elements software (NIKON). Images displayed are reconstructions of one z plane.

Real time-RT PCR. Total RNA was prepared using an RNAase kit (Qiagen), and mRNA expression levels were quantified using specific primers as listed in Table S2. Quantitative real time PCR was performed with SybrGreen on an Eppendorf PCR thermal cycler. Unless specified, β-actin was used for normalization. The relative fold change was calculated based on the $\Delta\Delta$Ct method.

Chromatin immunoprecipitation assay (ChIP). H9c2 cells were cultured with or without tunicamycin in serum-free media for 4 hours. Cells were treated with 1% formaldehyde for 10 min followed by glycine to terminate the cross-linking reaction. Approximately 7.5 x10^6 cells were lysed with ChIP lysis buffer according to the manufacturer's instructions (SimpleChIP Enzymatic Chromatin IP kit – magnetic beads, Cell Signaling). The chromatin was fragmented by incubation with 2000 gel units micrococcal nuclease per 7.5 x10^6 cells for 20 min at 37°C. The nuclear membrane was broken by 15 sec of gentle sonication and 5 µg of chromatin in 500 µl CHIP buffer was incubated overnight at 4°C with 5 µg normal rabbit IgG (#2729, Cell Signaling) or 5 µg anti-ATF4 Ab (#11815, Cell Signaling). ChIP grade Protein G magnetic beads (#9006) were used to precipitate attached chromatin. Eluted samples were subjected to reverse cross-linking by incubating with 250 µg/ml proteinase K at 65°C for 2 h. DNA was isolated and concentrated using DNA purification spin columns and eluted in 50 µl of elution buffer. The immunoprecipitated DNA was analyzed by semi-quantitative PCR using primers to detect the putative ATF4-binding sites in the Nox4 promoter. ATF4 primers were: forward TGGTCCTGACTTTTCCATCAG, reverse TGGATGTTCGAGAAATTGACTG. PCR was performed under standard conditions for 40 cycles with an annealing temperature of 55°C.

Immunoblotting. Cells were lysed in lysis buffer (composition: 10 mM HEPES pH 7.9, 50 mM NaCl, 0.5 M sucrose, 0.1 mM EDTA, 0.5% Triton X-100) containing phosphatase inhibitors (10 mM tetrasodium pyrophosphate, 100 mM NaF, 17 mM β-glycerophosphate, 1 mM sodium orthovanadate), a protease inhibitor cocktail (Sigma, #P8340) and a proteasome inhibitor (2 µg/ml Mg132). To quantify

phosphorylated proteins, media were removed and lysis buffer containing inhibitors was immediately added to protect phosphorylation sites. Nuclear proteins were extracted as previously described (Zhang et al, 2010). For caspase immunoblots, cells were harvested in culture medium, centrifuged at 2,800 *g* for 5 min, and the pellet resuspended in lysis buffer. Protein content was estimated by a Bradford Assay. Equal amounts of lysates or protein extracts were loaded onto SDS-polyacrylamide gels, and then transferred to nitrocellulose membranes. After blocking the membranes with Tris-buffered saline and 0.05% Tween 20 (TBST) containing 5% non-fat milk, the membranes were incubated overnight with primary antibodies at 4^oC. After incubating with horseradish peroxidase (HRP)-linked secondary antibodies, the blots were revealed by chemiluminescence. Antibodies used were: Nox4 (rabbit polyclonal,1:4,000) (Anilkumar et al, 2008); KDEL (Grp78 and Grp96) (1:10,000, Stressgene); phospho-eIF2α (1:4,000, Millipore); total eIF2α (1:2,000, Santa Cruz); phospho-Histone H3 (S10) (1:2,000, Millipore); total Histone H3 (1:2,000, Cell Signaling); phospho-PERK (1:2,000, Santa Cruz); total PERK (1:2,000, Santa Cruz); ATF4 (1:2,000, Santa Cruz); PP1 (1:2,000, Santa Cruz); rabbit GADD34 (1:2,000, Santa Cruz); mouse GADD34 (1:2,000, Novus Biological); phospho-glycogen synthase (Ser 641) (1:2,000, Cell Signaling); total glycogen synthase (1:2,000, Cell Signaling); PARP (1:2,000, Cell Signaling); caspase-3 (1:2,000, Cell Signaling); caspase-12 (1:2,000, Sigma Aldrich); ATF6 (1:2,000, Sigma Aldrich); FLAG (1:2,000, Sigma Aldrich); Myc (1:2,000, Santa Cruz); Calnexin (1:10,000, Sigma Aldrich); β-actin (1:2,000, Sigma Aldrich); tubulin (1:10,000, Sigma Aldrich); and GAPDH (1:10,000, Sigma Aldrich). Secondary antibodies used were: goat anti-rabbit-HRP (1:2,000, Sigma Aldrich), and goat anti-mouse-HRP (1:2,000, Sigma Aldrich). Densitometric analysis was performed using Image J software (NIH, USA).

Preparation of membrane fractions. Cells grown in 100-mm dishes were washed with cold PBS and homogenized in lysis buffer (50 mM Tris, pH 7.4, containing 0.1 mM EDTA, 0.1 mM EGTA, protease inhibitor cocktail [Sigma, #P8340], and 2 µg/ml Mg132) by sonication (10 s of 3 cycles at 8 W). After centrifugation at 18,000 g for 15 min to separate mitochondria and nuclei, the supernatant was further centrifuged at 100,000 g for 1 h. The resulting supernatant formed the cytosolic fraction. The pellet containing the membrane-enriched fraction was washed two times with the same lysis buffer to remove any remaining cytosol contamination. Membrane fractions were used to assay Nox activity, PP1 activity or for immunoblotting. The membrane fraction was enriched in the ER marker calnexin while the cytosolic fraction was enriched in GAPDH (Fig. S4A).

Sucrose gradient fractionation. H9c2 cells grown in 10 mm Petri dishes were scraped (4 dishes/sample), transferred into tubes and centrifuged at 2,800 *g* at 4°C for 5 min. The cell pellet was resuspended in 250 µl lysis buffer (50 mM Tris HCl pH 7.2, 150 mM NaCl, 2 mM EDTA, 0.5% Triton X-100 containing protease cocktail and Mg132 as described above). Cell lysates were laid at the top of sucrose gradient (10%, 20%, 40% and 60%, top to bottom), which was prepared in 50 mM HEPES buffer pH 7.5, containing 100 mM KCl, 2 mM $MgCl_2$, 1 mM EGTA and 1 mM EDTA. The samples were centrifuged at 35,000 *g* (4°C, 18 h). Fractions 1-16 (F1-F16) were collected from the base of the column. Each fraction was split into two 200 µl aliquots, one for immunoblotting and the other for immunoprecipitation experiments. As a control for density gradient separation, a mix of proteins (Gel filtration molecular weight markers, Sigma-Aldrich) was added to the top of the sucrose gradient in a

separate tube and centrifuged. The fractions obtained were submitted to SDS-PAGE and proteins were stained with Coomassie Blue.

Immunoprecipitation. Cells grown in 6 well plates were scraped and transferred into tubes, then centrifuged at 2,800 *g* at 4^oC for 5 min. The cell pellet was resuspended in 200 µl lysis buffer. Samples were briefly sonicated (one 10 s cycle, 8 W). Protein concentration was normalized to 1 µg/µl and immunoprecipitation (IP) was performed using 500 µg of homogenate protein. Protein A/G Sepharose beads (Santa Cruz Technology) were pre-cleared with nonspecific IgG, and samples were then precipitated overnight at 4^oC with specific antibody. Samples with non-specific antibody were used as negative control. The next day, immunoprecipitates were washed 7 times with buffer and resuspended in sample buffer for immunoblotting. Samples were heated at 95 °C for 5 min. After cooling, reducing agent was added, samples were run on SDS-polyacrylamide gels, and then blotted onto nitrocellulose. For co-immunoprecipitation with cMyc-tagged Nox4-constructs, IP was performed using an anti-cMyc immunoprecipitation kit (Sigma, IP 0020-IKT).

Measurement of ROS. Nox activity (NADPH-stimulated ROS generation) was measured in membrane fractions preapared as described above, using HPLC-based detection of the oxidation products of dihydroethidium (DHE, Invitrogen), i.e. 2-hydroxyethidine (EOH) and ethidium (E), as previously described (Fernandes et al, 2007; Laurindo et al, 2008). 20 µg of membrane protein was incubated with DHE (50 µM) at 37°C in the dark for different time periods after the addition of NADPH (300 µM) in PBS/DTPA.The reaction was stopped on ice until HPLC injection. For cellular ROS measurement, cells were washed with PBS and immediately incubated in 0.5

ml PBS/DTPA (0.1 mM) at a final DHE concentration of 100 µM for 30 min. The buffer was removed, cells washed with PBS/DTPA, harvested in cold acetonitrile (0.5 ml/well), sonicated (10 s, 1 cycle at 8 W), and centrifuged (12,000 *g* for 10 min at 4^0C). Supernatants were dried under vacuum. Pellets were stored at –20°C in the dark until analysis. Samples were resuspended in 120 µl PBS/DTPA and injected into a Dionex HPLC system. Quantification was performed by comparison of peak signal between the samples and standard solutions under identical chromatographic conditions (Laurindo et al, 2008). DHE-derived products were expressed as ratios of EOH and E generated per DHE consumed (initial DHE concentration minus remaining DHE; EOH/DHE and E/DHE).

ROS imaging in live cells. Intracellular ROS were visualized using HyPer probes, which are a fusion of the H_2O_2-sensitive domain of the *E. Coli* transcription factor OxyR with a fluorescent protein for signal detection (Belousov et al, 2006; Ermakova et al, 2014). The Cys199 residue in Hyper proteins is highly susceptible to oxidation by H_2O_2, resulting in a change in protein conformation and in fluorescence that can be visualized by imaging. Furthermore, Hyper proteins can be targeted to different cell compartments such as the ER and cytosol. The use of a conventional HyPer probe emitting green fluorescence in combination with the recently developed HyperRed probe, which emits red fluorescence, allows the simultaneous imaging of ROS in two subcellular compartments. We used a HyPer probe targeted to the ER with a C-terminal KDEL sequence (Hyper-ER) together with a cytosolic HyPerRed probe (Enyedi et al, 2010; Ermakova et al, 2014). HyPer-ER was a kind gift from Dr Miklós Geiszt (Department of Physiology, Semmelweis University, Budapest, Hungary). The respective C199S mutant probes for HyPer-ER and HyPerRed, which

are ROS-insensitive, were used as negative controls to exclude changes in pH. Cells were co-transfected with HyPer-ER and HyPerRed and kept in phenol red-free medium supplemented with 2 mM glutamine and antibiotics for 48 hours before treatment with tunicamycin (2 µg/ml for 4 hours) or control vehicle. Imaging was performed at 37° C / 5% CO_2 on an inverted Nikon Ti-E microscope equipped with a Yokogawa CSU-X1 spinning-disk confocal unit, an Andor Neo sCMOS camera and a Sutter filter wheel. A 60x Plan Apo *VC* NA 1.40 Nikon objective was used. HyPer-ER fluorescence emission was monitored at 525/50 nm following excitation at 405 nm and 488 nm, and the ratio of fluorescence intensity was quantified. HyPerRed fluorescence emission was monitored at 647/75 nm following excitation at 560/40 nm. Extracellular H_2O_2 (200 nM) was added as a positive control and the HyPer-ER and HyPerRed signals acquired simultaneously. NIS Elements v.4.0 software (Nikon) was used for image analysis. Images were background-subtracted and thresholded. Changes in HyPer-ER fluorescence ratio (ΔR) or HyPerRed fluorescence intensity (ΔF) between the indicated time-points or treatments were quantified. The resulting images were displayed in pseudocolor.

Recombinant PP1 expression and purification. A pCW vector expressing the untagged γ isoform of the catalytic subunit of human PP1 (Alessi et al, 1993) was obtained from the MRC Protein Phosphorylation Unit (Dundee, UK). Protein expression and purification was carried out essentially as described (Barford and Keller, 1994; Egloff et al, 1995). Transformed *E.coli* DH5α cells were grown in Luria-Bertani (LB) medium supplemented with 2 mM $MnCl_2$ and 100 µg.ml^{-1} ampicillin at 30 °C until OD600 reached approximately 0.25. Protein expression was induced with 0.5 mM IPTG. Cells were harvested by centrifugation at 5000 g for 15 min at 4 °C

and resuspended in buffer A (50 mM imidazole, 0.5 mM EDTA, 0.5 mM EGTA, 100 mM NaCl, 10% glycerol, 2 mM β-mercaptoethanol, 2 mM $MnCl_2$, pH 7.5) supplemented with Complete EDTA-free protease inhibitor cocktail (Roche), lysozyme (0.01 mg/ml) and DNAse (0.05 mg/ml). Cell lysis was accomplished by sonication or using a cell disruptor (Constant Systems Ltd). Insoluble material was sedimented by centrifugation at 19500 g for 1 h at 4 °C and the supernatant filtered using 0.22 μm prior to loading on a 5 mL heparin column equilibrated with buffer A. PP1 was eluted using a 100 ml gradient to 50% buffer A supplemented with 1M NaCl. Fractions were analysed on a 12% SDS-PAGE gel and those containing PP1 were pooled and diluted 10-fold with buffer C (50 mM imidazole, 0.5 mM EDTA, 0.5 mM EGTA, 10% glycerol, 5 mM β-mercaptoethanol, 2 mM $MnCl_2$, pH 7.2) for injection in a HiTrapQ HP (GE Healhcare) column. PP1 was eluted using a gradient to 40% buffer C supplemented with 1M NaCl. PP1 was further purified by size-exclusion chromatography (SEC) using a Superdex 75 16/60 (GE Healthcare) column equilibrated with SEC buffer (50 mM imidazole, 0.5 mM EDTA, 0.5 mM EGTA, 300 mM NaCl, 10% glycerol, 5 mM β-mercaptoethanol, 2 mM $MnCl_2$, pH 7.5) for downstream applications. PP1 mutations (PP1 N124D and PP1 D64N) were introduced using the Q5 Site-Directed Mutagenesis Kit (New England Biolabs). All constructs were verified by sequencing. Expression and purification of PP1 variants were carried out as for wild-type PP1.

Crystal Preparation, data collection and structure solution. PP1 at 4.5 mg/ml in SEC buffer was used to set up crystallization drops using the vapor diffusion method at 18 °C. Crystals belonging to the space group $P2_1$ grew either in 20% PEG2000 MME, 200 mM NaCl, 0.1 M Tris-HCl pH 9.0 (ref. 28) or in 7-12% PEG3350, 0.1 M

Bicine, pH 9.0. To ensure the reduced state of the PP1 dinuclear center, PP1 crystals were soaked with a reservoir solution enriched with 25 mM sodium ascorbate. PP1 oxidation was carried out by soaking PP1 crystals in crystallization reservoir spiked with 50 mM H_2O_2 for 10 min. Crystals were cryoprotected by soaking them in their respective reservoir solutions supplemented with either 20% glycerol or 25% 2-methyl-2,4-pentanediol (MPD) for a few seconds. Datasets for ascorbate-treated PP1 (reduced-PP1) and H_2O_2-treated PP1 (oxidized-PP1) crystals were collected at the Diamond Light Source (Oxford, UK) at beamlines I04-1 (reduced-PP1) and I03 (oxidized-PP1). Synchrotron data were processed with the xia2 pipeline (Winter, 2010; Kabsch, 2010). Model coordinates have been deposited with the Protein Data Bank with codes 4UT2 and 4UT3 for ascorbate-treated and hydrogen peroxide-treated PP1, respectively. The structure of PP1 was solved by the molecular replacement technique using the program MOLREP (Vagin and Teplyakov, 1997) starting from the coordinates of rat PP1 (PDB code 2O8A) as search model (Hurley et al, 2007). The model was subsequently completed manually using Coot (Emsley and Cowtan, 2004) and refined using Refmac5 (Murshudov et al, 2011; Steiner et al, 2003) without restraints for metal-ligand distances. As metal analysis (*vide infra*) indicated that Mn is approximately six times more abundant than Fe in the crystal and anomalous electron density maps calculated from data collected at 6876.6 eV identified Mn ions at both M1 and M2 sites, we refined both metal centers as Mn. An attempt to specifically locate Fe ions using a double difference anomalous map approach (Than et al, 2005) was unsuccessful likely owing to the low Fe content. A summary of data collection and refinement statistics are shown in Table S3.

Metal analysis. Metal content of PP1 crystals was assessed by collecting a fluorescence spectrum using an X-ray excitation energy of 18 keV. Fitting was performed with the PyMCA package (Solé et al, 2007).

PP1 reactions and activity. All buffers were pretreated with Chelex-100 to remove transition metal ion contamination. Dialysis and concentration of the different recombinant PP1 proteins was performed in 0.1 M Tris-HCl buffer, pH 7.0, at 4°C, using Amicon Ultra 10 k centrifugal filters (Millipore). PP1 protein concentration was measured using a Nanodrop 2000 (Thermoscientific) and protein was immediately used for the oxidation reaction experiments. In some experiments, we used recombinant PP1α purchased from Sigma (# P7937-25UG). To study the effects of ROS, the reaction mixture containing PP1 (17 µg/ml) was treated with different concentrations of H_2O_2 for 15 min at 37°C and then further treated with catalase (30 U/ml) for 15 min at room temperature to remove residual H_2O_2. In experiments with DTT, cysteine, GSH, these were added at a concentration of 0.5 mM and 1 mM each for 30 min. Ascorbate was added for 30 min at the concentrations indicated in the figures. To measure activity, samples were incubated at 37°C in the absence or presence of 40 µM phospho-threonine peptide (Lysine-Arginine-phosphoThreonine-Isoleucine-Arginine-Argine; K-R-pT-I-R-R) (Millipore). After 15 min, samples were incubated with 0.1 ml of Malachite Green reagent (Millipore, #17-127) and the hydrolysis of the phosphopeptide was measured spectrophotometrically at 620 nm using a Nanodrop spectrophotometer, according to the manufacturer's instructions.

For assessment of cellular phosphatase activity, cell membrane extracts (Hubbard et al, 1990) were incubated with phosphopeptide substrate (0.1 mM) in the presence or absence of okadaic acid (10 nM), which does not inhibit PP1 at this

concentration (Ishihara et al, 1989), and then phosphatase activity was estimated as described above. For each sample, incubation without the phosphopeptide substrate was used as a blank. PP1 activity was taken as the okadaic acid-resistant fraction and was normalized by protein content. Calyculin A (60 nM) (which inhibits both PP1 and PP2a) (Ishihara et al, 1989) was used as a control to confirm total PP activity. In some experiments, ascorbate (0.5 mM) was added to cells for 30 min before cell lysis.

Electron paramagnetic resonance spectroscopy (EPR). EPR was used to measure ascorbyl radical generation and to assess the PP1 metal redox status. For ascorbyl detection, EPR spectra were recorded at room temperature in a Magnatech Miniscope MS2000 spectrometer. The instrument conditions were: microwave power 50 mW, modulation amplitude 1 Gauss (G), scan time 328 ms, with a gain of 9×10^2. All spectra were the accumulation of 4 scans and were recorded 5 min after addition of H_2O_2. EPR instrument conditions were calibrated with 4-hydroxy-2, 2, 6, 6-tetramethyl-1-piperidinyloxy (Tempol). The reaction was carried out in 0.1 mM Tris buffer at pH 7.0 and 37 ^oC under the different conditions described in Figure legends, and was transferred to a 50 µl flat cell immediately after the addition of ascorbate. The two line spectrum was consistent with an ascorbyl radical with a hyperfine splitting constant (a_H = 1.8G) (Monteiro et al, 2007), as generated using the positive control ascorbate and H_2O_2.

EPR at low temperature is a method to detect chemical species with unpaired electrons and is used for studies of transitional metal ion complexes in proteins (Cammack and Cooper, 1993; Ubbink et al, 2002). We used a Bruker EMX 300 spectrometer with a 3mm cavity and a helium cooling system. Purified PP1 (5 mg/ml)

was studied at baseline and after treatment with H_2O_2 (1 mM) in TrisHCl buffer pH 7.2 at 37°C. The reaction mixture was transferred to a flat cell and frozen in liquid nitrogen. Spectrometer conditions were: temperature, 4 K; microwave frequency, 9.66 GHz; modulation amplitude, 2 G at 100 kHz; microwave power, 20mW.

Cell viability. Cells were plated in 24-well plates at 70% confluence and Nox4 levels were manipulated as described in the Figure legends. Cells were then exposed to tunicamycin (2 µg/ml), guanabenz or clonidine (both dissolved in PBS), or salubrinal (dissolved in DMSO) at the indicated concentrations. A DMSO-alone group was employed as a vehicle control. Cell viability was assessed by measuring the reduction of yellow MTT (3-(4,5-dimethylthiazol-2-yl)-2,5-diphenyltetrazolium bromide) into purple formazan. Briefly, MTT solution (1.25 mg/ml dissolved in PBS) was sonicated and added. After 2 h, the medium was gently removed and MTT solvent solution (isopropyl alcohol containing 0.1% NP40 and 4 mM HCl) was added. After mixing for 30 min at room temperature in the dark, the absorbance was measured at 550 nm using a Nanodrop 2000 spectrophotometer (Thermoscientific).

Animal studies. All procedures were performed in compliance with the UK Home Office 'Guidance on the Operation of the Animals' (Scientific Procedures) Act, 1986 and institutional guidelines. Nox4$^{-/-}$ mice on a C57BL6 background were described previously (Zhang et al, 2010). We used 8-12 week old male Nox4$^{-/-}$ mice and matched WT littermates. Animals were maintained in a registered Biological Services Facility under approved husbandry conditions and standardized light/dark cycles.

Heart I/R injury was assessed in *ex-vivo* hearts perfused on a Langendorff system with a modified Krebs-Henseleit (KH) buffer containing 10 mM glucose and

0.4 mM octanoate. A constant perfusion pressure of 70 mmHg was used at 37°C. After 20 min equilibration, global ischemia was initiated for 25 min and the hearts were then reperfused for 100 min. Hearts were weighed, frozen and cut into 1 mm thick slices. Viable tissue was stained red with 1% 2,3,5-triphenyl-tetrazolium chloride (TTC) in phosphate buffer; sections were then immersed in formalin and scanned. The infarcted area was calculated as a proportion of the total left ventricular area using Image J Software. For immunobloting studies, hearts were reperfused for 30 min following ischemia and snap frozen for subsequent analyses. Some animals were injected with guanabenz (1.8 mg/kg body weight) 24 h prior to heart perfusion. The hearts of these animals were perfused with modified KH buffer containing 0.5 µM guanabenz.

To induce ER stress-related AKI, animals were treated with tunicamycin (3 mg/kg/day i.p. for two days) (Zinszner et al, 1998). Some animals were pre-treated with guanabenz (1.8 mg/kg ip). After sacrifice, serum was collected and the plasma urea concentration was measured using a commercial Kit (Bioassay Systems). Kidneys were harvested for immunoblotting or were fixed and paraffin-embedded to assess apoptosis using TUNEL staining (Millipore S7110 Kit).

Statistics. Data are presented as mean±SEM. Comparisons among groups were undertaken by Student's t test or one-way ANOVA, as appropriate. Kaplan Meier analysis was used to compare survival. Statistical analyses were performed on GraphPad-Prism (GraphPad-Software, San Diego, Ca). $P<0.05$ was considered significant.

References

Alessi DR, Street AJ, Cohen P, Cohen PT (1993) Inhibitor-2 functions like a chaperone to fold three expressed isoforms of mammalian protein phosphatase-1 into a conformation with the specificity and regulatory properties of the native enzyme. Eur J Biochem 213: 1055-1066

Anilkumar N, Weber R, Zhang M, Brewer A, Shah AM (2008) Nox4 and Nox2 NADPH oxidases mediate distinct cellular redox signaling responses to agonist stimulation. Arterioscler Thromb Vasc Biol 28: 1347-1354

Anilkumar N, San Jose G, Sawyer I, Santos CX, Sand C, Brewer AC, Warren D, Shah AM (2013) A 28-kDa splice variant of NADPH oxidase-4 is nuclear-localized and involved in redox signaling in vascular cells. Arterioscler Thromb Vasc Biol 33: e104-e112

Barford D, Keller JC (1994) Co-crystallization of the catalytic subunit of the serine/threonine specific protein phosphatase 1 from human in complex with microcystin LR. J Mol Biol 235: 763-766

Belousov VV, Fradkov AF, Lukyanov KA, Staroverov DB, Shakhbazov KS, Terskikh AV, Lukyanov S (2006) Genetically encoded fluorescent indicator for intracellular hydrogen peroxide. Nature Methods 3, 281-286

Cammack R, Cooper CE (1993) Electron paramagnetic spectroscopy of iron complexes and iron-containing proteins. Methods Enzymol 227: 353–384

Debeurme F, Picciocchi A, Dagher MC, Grunwald D, Beaumel S, Fieschi F, Stasia MJ (2010) Regulation of NADPH oxidase activity in phagocytes: relationship between FAD/NADPH binding and oxidase complex assembly. J Biol Chem 285: 33197-33208

Dinauer MC, Curnutte JT, Rosen H, Orkin SH (1989) A missense mutation in the neutrophil cytochrome b heavy chain in cytochrome-positive X-linked chronic granulomatous disease. J Clin Invest 84: 2012-2016

Egloff MP, Cohen PT, Reinemer P, Barford D (1995) Crystal structure of the catalytic subunit of human protein phosphatase 1 and its complex with tungstate. J Mol Biol 254: 942-959

Emsley P, Cowtan K (2004) Coot: Model-building tools for molecular graphics. Acta Crystallogr D ***60***: 2126-2132

Enyedi B, Várnai P, Geiszt M (2010) Redox state of the endoplasmic reticulum is controlled by Ero1L-alpha and intraluminal calcium. Antioxid Redox Signal 13: 721-729

Ermakova YG, Bilan DS, Matlashov ME, Mishina NM, Markvicheva KN, Subach OM, Subach FV, Bogeski I, Hoth M, Enikolopov G, Belousov VV (2014) Red fluorescent genetically encoded indicator for intracellular hydrogen peroxide. Nat Commun 5: 5222 doi: 10.1038/ncomms6222

Fernandes DC, Wosniak J Jr, Pescatore LA, Bertoline MA, Liberman M, Laurindo FR, Santos CX (2007) Analysis of DHE-derived oxidation products by HPLC in the assessment of superoxide production and NADPH oxidase activity in vascular systems. Am J Physiol 292: C413-C422

Hubbard MJ, Dent P, Smythe C, Cohen P (1990) Targetting of protein phosphatase 1 to the sarcoplasmic reticulum of rabbit skeletal muscle by a protein that is very similar or identical to the G subunit that directs the enzyme to glycogen. Eur J Biochem 189: 243-249

Hurley TD, Yang J, Zhang L, Goodwin KD, Zou Q, Cortese M, Dunker AK, DePaoli-Roach AA (2007) Structural basis for regulation of protein phosphatase 1 by inhibitor-2. J Biol Chem 282: 28874-28883

Ishihara H, Martin BL, Brautigan DL, Karaki H, Ozaki H, Kato Y, Fusetani N, Watabe S, Hashimoto K, Uemura D, Hartshorne DJ (1989) Calyculin A and okadaic acid: inhibitors of protein phosphatase activity. Biochem Biophys Res Commun 159: 871-877

Kabsch W (2010) XDS. Acta Crystallogr D Biol Crystallogr 66: 125-132

Laurindo FR, Fernandes DC, Santos CX (2008) Assessment of superoxide production and NADPH oxidase activity by HPLC analysis of dihydroethidium oxidation products. Methods Enzymol 441: 237-260

Monteiro G, Horta BB, Pimenta DC, Augusto O, Netto LE (2007) Reduction of 1-Cys peroxiredoxins by ascorbate changes the thiol-specific antioxidant paradigm, revealing another function of vitamin C. Proc Natl Acad Sci USA 104: 4886-4891

Murshudov GN, Skubak P, Lebedev AA, Pannu NS, Steiner RA, Nicholls RA, Winn MD, Long F, Vagin AA (2011) REFMAC5 for the refinement of macromolecular crystal structures. Acta Crystallogr D 67: 355-367

Peterson JR, Burmeister MA, Tian X, Zhou Y, Guruju MR, Stupinski JA, Sharma RV, Davisson RL (2009) Genetic silencing of Nox2 and Nox4 reveals differential roles of these NADPH oxidase homologues in the vasopressor and dipsogenic effects of brain angiotensin II. Hypertension 54: 1106-1114

Solé VA, Papillon E, Cotte M, Walter Ph, Susini J (2007) A multiplatform code for the analysis of energy-dispersive X-ray fluorescence spectra. Spectrochimica Acta Part B 62: 63-68

Steiner RA, Lebedev AA, Murshudov GN (2003) Fisher's information in maximum-likelihood macromolecular crystallographic refinement. Acta Crystallogr D 59: 2114-2124

Than ME, Henrich S, Bourenkov GP, Bartunik HD, Huber R, Bode W (2005) The endoproteinase furin two essential Ca2+ ions stabilizing its N-terminus and the unique S1 specificity pocket. Acta Crystallogr D 61: 505-512

Ubbink M, Worrall JA, Canters GW, Groenen EJ, Huber M (2002) Paramagnetic resonance of biological metal centers. Annu Rev Biophys Biomol Struct 31: 393–422

Vagin A, Teplyakov A (1997) MOLREP: An automated program for molecular replacement. J Appl Crystallogr 30: 1022-1025

Winter G (2010) xia2: an expert system for macromolecular crystallography data reduction. J Appl Crystallogr 43: 186-190

Zhang H, Ma Y, Liu K, Yu JG (2013)Theoretical studies on the reaction mechanism of PP1 and the effects of different oxidation states of the Mn-Mn center on the mechanism. J Biol Inorg Chem 18: 451-459

Zhang M, Brewer AC, Schröder K, Santos CX, Grieve DJ, Wang M, Anilkumar N, Yu B, Dong X, Walker SJ, Brandes RP, Shah AM (2010) NADPH oxidase-4 mediates protection against chronic load-induced stress in mouse hearts by enhancing angiogenesis. Proc Natl Acad Sci USA 107: 18121-18126

Zinszner H, Kuroda M, Wang X, Batchvarova N, Lightfoot RT, Remotti H, Stevens JL, Ron D (1998) CHOP is implicated in programmed cell death in response to impaired function of the endoplasmic reticulum. Genes Dev 12: 982-995

APPENDIX FIGURE LEGENDS

Figure S1. Nox4 levels and Nox4-dependent ROS production increase during protein unfolding stress.

A. Effect of tunicamycin (Tn) on Nox4 mRNA levels in H9c2 cells.

B. Nox4 mRNA levels in rat primary cardiomyocytes were increased in response to Tn treatment in a time- and dose-dependent manner.

C. Ad.shNox4 (shRNA-mediated knockdown of Nox4) significantly reduced Nox4 mRNA levels in H9c2 cells, both at baseline and after tunicamycin treatment.

D. Nox activity increased after tunicamycin treatment but was substantially reduced by the knockdown of Nox4. Nox activity was measured in membrane fractions isolated after 4h of Tn treatment, using HPLC-based detection of the dihydroethidium (DHE) oxidation products, 2-hydroxyethidium (EOH) and ethidium (E). Inset shows Nox4 protein levels.

E. Effects of shRNA-mediated knockdown of Nox4 in H9c2 cells on the tunicamycin (Tn, 2 µg/ml)-induced changes in nuclear levels of ATF4 (red bar graph) and ATF6 (black bar graph); mRNA levels of *Xbp1-s* (blue bar graph); and protein levels of Grp78 (green bar graph). Representative blots and gels for this experiment are shown in Fig 1C.

All data are mean ± SEM of n=3 per group except panel D, which is n=4 per group. *, significant compared to baseline. #, significant comparing Ad.shNox4 versus corresponding control (Ad.Ctl). Values above bar graphs denote the level of significance.

Figure S2. Nox4 selectively regulates the ATF4 limb of the UPR during ER stress.

A. Effect of siRNA-mediated knockdown of Nox4 (siNox4) on the UPR in tunicamycin (Tn, 2 µg/ml)-stimulated H9c2 cells. siCtl denotes treatment of cells with a control scrambled siRNA. Mean changes in nuclear levels of ATF4 and ATF6; mRNA levels of *Xbp1-s*; and protein levels of Grp78 are shown to the right. Representative blots are shown to the left.

B. Quantification of changes in UPR readouts in H9c2 cells subjected to adenoviral-mediated Nox4 overexpression (Ad.Nox4) or a control vector (Ad.β-Gal). The same UPR readouts were studied as in panel A. Representative blots and gels for this experiment are shown in Fig 1D.

C. *Nox4* mRNA levels in H9c2 cells after siRNA-mediated silencing of ATF4 as in Fig 1G.

D. Chromatin immunoprecipitation (ChIP) analyses to detect *in vivo* association of ATF4 to the Nox4 promoter region in H9c2 cells. Chromatin from H9c2 cells treated with tunicamycin or control vehicle was immunoprecipitated with an anti-ATF4 antibody or with non-specific rabbit IgG, as indicated. Purified DNA was analyzed using primers specific for the rat *Nox4* promoter comprising the putative ATF4 binding sites (see schematic).

All data are mean ± SEM of 3 experiments/group apart from ATF6 protein levels in panel A which were n=4/group. *, significant compared to baseline. #, significant comparing siNox4, Ad.Nox4 or siATF4 versus corresponding controls. Values above bar graphs denote the level of significance.

Figure S3. Effects of Nox4 on UPR readouts and PP1 targets. Quantitative data are shown corresponding to the representative immunoblots in Fig 2.

A-E. **C**hanges in protein levels of phosphorylated PERK (PERK-P), GADD34, PP1, phosphorylated glycogen synthase (GS-P), and phosphorylated Histone H3 (H3-P) in tunicamycin-treated H9c2 cells with knockdown of Nox4 (Ad.shRNA) as compared to control (Ad.Ctl). Representative blots are shown in Fig 2A and 2G.

F-J. Analogous data for H9c2 cells with overexpression of Nox4 (Ad.Nox4) or a control (Ad.β-Gal). Representative blots are shown in Fig 2B and 2H.

K-O. Response to tunicamycin of Nox4$^{-/-}$ MEFs (KO), WT MEFs or Nox4$^{-/-}$ MEFs in which Nox4 was reintroduced (KO+Nox4). Representative blots are shown in Fig 2I. All data are mean ± SEM of 4 experiments/group apart from panels B,F,G,J and O which were n=3/group. *p<0.05, **p<0.01 cf. baseline; #, p<0.05, comparing Ad.shNox4 or Ad.Nox4 versus corresponding control, or KO versus WT and KO+Nox4.

Figure S4. Association of Nox4 with GADD34.

A. Membrane fractions of H9c2 cells are enriched in the ER marker calnexin while the cytosolic fraction is enriched in GAPDH protein

B. Sucrose density gradient separation of a mixture of immunoglobulin M (IgM), bovine liver catalase (BLC) and cytochrome C (CytoC) as control proteins of known molecular weight. The protein mixture was added to the top of the sucrose gradient. After centrifugation, the fractions were collected and submitted to SDS-PAGE and proteins were stained with Coomassie Blue. The graph depicts the densitometry values of each control protein obtained in the individual fractions.

C. H9c2 cells were infected with Ad.Nox4, then treated with tunicamycin (2 µg/ml, 6 hours). Total cell lysates were subjected to sucrose gradient fractionation. The immunoblot shows that Nox4 eluted in fractions 12 and 13 (F12, F13).

D. Confocal microscopic images of MEFs transfected with Myc-tagged HyPer ER and stained for Myc (red) and PDI (green) as an ER marker. The merged image shows co-localization of HyPer ER and PDI (yellow); a higher magnification of the inset is shown to the right. Scale bars, 5 µm.

E. Confocal microscopic images of WT MEF cells co-transfected with HyPer ER and HyPerRed Cyto. The distinct localization of the two probes is evident from the lack of yellow color; a higher magnification of the inset is shown at the bottom. Scale bars 5 µm.

Figure S5. Nox4 enhances cell survival.

A. Representative micrographs of H9c2 cells treated with tunicamycin (2 µg/ml, 48h), showing significantly lower survival when endogenous Nox4 was silenced (siNox4) as compared to cells treated with a scrambled siRNA (siCtl). Cell survival rates were normalized by treatment with guanabenz (Gbz, 5 µM).

B. Immunoblotting showed that cells in which Nox4 was depleted had higher levels of cleaved caspase-3 after tunicamycin treatment than cells treated with a scrambled siRNA. Levels of cleaved caspase-3 were reduced by treatment with guanabenz (Gbz, 5 µM) or salubrinal (Sal, 50 µM). Tubulin was used a loading control.

C. Densitometry values related to panel B. Data are means ± SEM of 3 independent experiments per group. *, significant comparing siNox4 versus corresponding siCtl. #, significant for effect of Gbz or Sal.

Table S1. Metal coordination distances in Å.

	Ascorbate-PP1* Reduced	H_2O_2-PP1* Oxidized	Theory Mn(II)-Mn(II)**	Theory Mn(III)-Mn(III)**
M1-M2	3.32/3.37 | (3.35)	3.35/3.33 | (3.34)	-	
M1-D64(OD2)	2.10/2.08 | (2.09)	1.96/1.99 | (1.98)	2.02	1.81
M1-H66(NE2)	2.24/2.36 | (2.30)	2.22/2.25 | (2.24)	2.38	2.31
M1-D92(OD2)	2.34/2.49 | (2.42)	2.06/2.04 | (2.05)	2.37	1.93
M1-W	2.44/2.07 | (2.26)	1.96/2.11 | (2.04)	2.38	2.13
M1-PO4	2.39/2.35 | (2.37)	1.99/2.25 | (2.12)	2.26	1.94
M1-OH	2.02/1.99 | (2.01)	2.23/2.20 | (2.22)	2.07	2.33
M2-D92(OD2)	2.31/2.23 | (2.27)	2.22/2.22 | (2.22)	2.31	2.88
M2-N124(OD1)	2.15/2.10 | (2.13)	1.89/1.94 | (1.92)	2.18	1.95
M2-H173(NE2)	2.36/2.19 | (2.28)	2.24/2.15 | (2.20)	2.31	2.11
M2-H248(ND1)	2.18/2.23 | (2.21)	2.32/2.32 | (2.32)	2.40	2.23
M2-PO4	2.04/2.48 | (2.26)	2.17/2.17 | (2.17)	2.50	1.98
M2-OH	2.06/2.04 | (2.05)	1.79/1.81 | (1.80)	2.02	1.84
M1+M2 coordination average	2.22/2.22 | (*2.22*)	2.09/2.12 | (***2.10***)	*2.27*	***2.12***
M1 coordination average	2.26/2.22 | (*2.24*)	2.07/2.14 | (***2.11***)	*2.25*	***2.08***
M2 coordination average	2.18/2.21 | (*2.20*)	2.11/2.10 | (***2.10***)	*2.29*	***2.17***

* First and second values refer to the two independent PP1 molecules in the a.u. The third value is their average. ** Taken from Zhang et al, 2013.

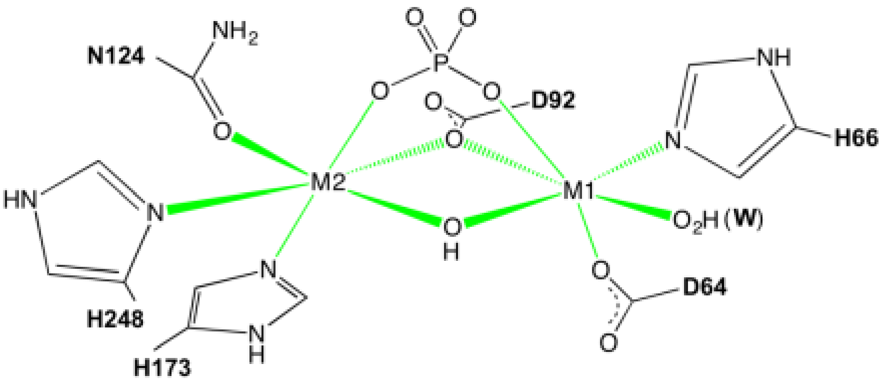

The maximum likelihood estimate for coordinates' uncertainty (σ_x) derived from crystallographic refinement is 0.08 Å and 0.11 Å for the ascobate-treated (reduced) and H_2O_2-treated (oxidized) structures, respectively. The standard uncertainty on metal-ligand (M-L) distances is $\sigma_{d(M\text{-}L)} = 2^{(1/2)}\sigma_x$. As there are two PP1 molecules in the a.u., each distance is determined twice resulting in the standard estimate of the mean (s.e.m.) to be (s.e.m. $d_{M\text{-}L}) = (2^{(1/2)}\sigma_x) / 2^{(1/2)} = \sigma_x$. Thus, s.e.m $d_{(M\text{-}L)reduced} = 0.08$ Å and s.e.m $d_{(M\text{-}L)oxidized} = 0.11$ Å.

To evaluate whether a correlation exists between the change in metal-ligand coordination distance defined as $\Delta = d_{(M\text{-}L)oxidized} - d_{(M\text{-}L)reduced}$ determined by theoretical and X-ray experimental methods we plotted $\Delta_{X\text{-ray}}$ against Δ_{theory} for each of the twelve (M-L) distances. A perfect correlation would result in a straight line of unitary slope. Errors for $\Delta_{X\text{-ray}}$ are calculated as $\Delta_{X\text{-ray}} = [(\sigma_{x(oxidised)})^2 + (\sigma_{x(reduced)})^2]^{(1/2)}$. The plot is shown in Fig. S5f.

There is a good correlation between theory and experiment with 75% of the Δ values (black circles) lying on the diagonal within error whilst three values (red circles) can be considered outliers. The availability of X-ray data at higher resolution and the use of a more complex description of protein restraints in the theoretical calculations might improve the agreement even further. The correlation is statistically significant and quantified by a Spearman ρ coefficient of 0.706 for all twelve Δ values with a two-tailed p-value of 0.0124.

The plot shows that most points lie on the lower-left quadrant. This implies a contraction of the average (M-L) distance upon metal oxidation. We tested whether the contraction of the average (M-L) distance observed experimentally is statistically significant. A Wilcoxon matched-pairs signed rank test gives a one-tailed p-value of 0.023 indicating statistical significance ($p<0.05$).

Table S2: List of cDNA primers

Gene name	Source	Sequence
Mouse XBP1 (total)	Sigma	F: 5’-GGCCTTGTGGTTGAGAACCAGGAG-3’<br>R: 5’-GAATGCCCAAAAGGATATCAGACTC-3’
Mouse β-actin	Sigma	F: 5’-CTGTCGAGTCGCGTCCACCC-3’<br>R: 5’-ATGCCGGAGCCGTTGTCGAC-3’
Mouse XBP1s	Sigma	F: 5’-AAGAACACGCTTGGGAATTGG-3’<br>R: 5’-CTGCACCTGCTGCGGAC-3’
Mouse XBP1 (total)	Sigma	F: 5’-CCTGAGCCCGGAGGAGAA-3’<br>R: 5’-CTCGAGCAGTCTGCGCTG-3’
Mouse Nox4	Sigma	F: 5’-CCGGACAGTCCTGCTTATC-3’<br>R: 5’-TGCTTTTATCCAACAATCTTCT-3’
Mouse pSAT1	Sigma	F: 5’-AGTGCCCATCAGTCCTTGAC-3’<br>R: 5’-GCCGTTGTTCTTGATCCATT-3’
Mouse SLC6A9	Sigma	F: 5’-CTGTCTGGCAACCTGTCTCA-3’<br>R: 5’-TCAGCACATACAGCCTCCAG-3’
Mouse PHDH	Sigma	F:5`-CAACCCCTGCTCGTATTCC-3`<br>R:5`-TGGAGGTTTGGTAGGACAGC-3`
Mouse ASNS	Sigma	F: 5’-TTCCTCACTCTTATCGGcTGC-3’<br>R: 5’-TGCCTGTGGTCCACCTTCTC-3’
Rat β-actin	Sigma	F: 5’-CCCGCGAGTACAACCTTCT-3’<br>R: 5’-CGTCATCCATGGCGAACT-3’
Rat XBP1s	Sigma	F: 5’-AAACAGAGTAGCAGCACAGACTGC-3’<br>R: 5’-TCCTTCTGGGTAGACCTCTGGGAG-3’
Rat XBP1 (total)	Sigma	F: 5’-GATGAATGCCCTGGTTACTG-3’<br>R: 5’-AGATGTTCTGGGGAGGTGAC-3’
Rat Nox4	Sigma	F: 5’-AGCTCATTTCCCACAGACCT-3’<br>R: 5’-TCCGGATGCATCGGTAAAGT-3’
Rat ATF4	Sigma	F: 5’-GAATGGCTGGCTATGGATGG-3’<br>R: 5’-AACATCCAATCTGTCCCGGA-3’
Rat pSAT1	Sigma	F: 5’-GGTCACCAGATTTCGCCAAG-3’<br>R: 5’-ATTTAAGGGGACGGCACTGA-3’
Rat SLC6A9	Sigma	F: 5’-CTGGGTGGTTGTCTTCCTCT-3’<br>R: 5’-CATAGGGAAATGTGGCCGTG-3’
Rat PHDH	Sigma	F: 5’-GGAAATAGCCTCAGTGCTGC-3’<br>R: 5’-CGGTCCCATTTGCCATCTTT-3’
Rat ASNS	Sigma	F: 5’-AAACGCTTGATGACTGACCG-3’<br>R: 5’-GCCTCCTTGAGTTGCTTCAG-3’

Table S3. X-ray crystallographic data collection and refinement statistics.

Data set	Ascorbate-treated PP1 (Reduced PP1)	H_2O_2-treated PP1 (Oxidised PP1)
**Data collection**		
Beam Line	I04-1 (DLS)	I03 (DLS)
Wavelength (Å)	0.9200	
Resolution range (Å)<br>Highest res. bin (Å)	64.12-1.96<br>(2.01-1.96)	45.40-2.20<br>(2.26-2.20)
Space group	$P2_1$	$P2_1$
Cell dimensions<br>a, b, c (Å)<br>β (°)	 38.55, 90.24, 91.13<br>90.05	 38.45, 105.22, 89.82<br>90.34
Unique reflections	42357<br>(3158)	35328<br>(2624)
Overall redundancy	4.0<br>(4.0)	3.3<br>(3.3)
Completeness, (%)	94.7<br>(95.6)	97.5<br>(97.3)
R_{symm}, (%)	7.8<br>(60.8)	8.1<br>(61.5)
$\langle I/\sigma(I) \rangle$	11.7<br>(2.5)	7.9<br>(2.1)
**Refinement**		
PDB code	4UT2	4UT3
R_{factor} (%)/R_{free} (%)	16.7/20.1	17.3/21.1
Twinning fraction (%)	55.5/44.5	90.2/9.8
Twinning operator	-H, -K, L	H, -K, -L
# non H-atoms	5002	4936
rms bond lengths (Å)	0.005	0.009
rms bond angles (°)	0.998	1.267
Wilson *B* value (Å^2)	25.6	38.3

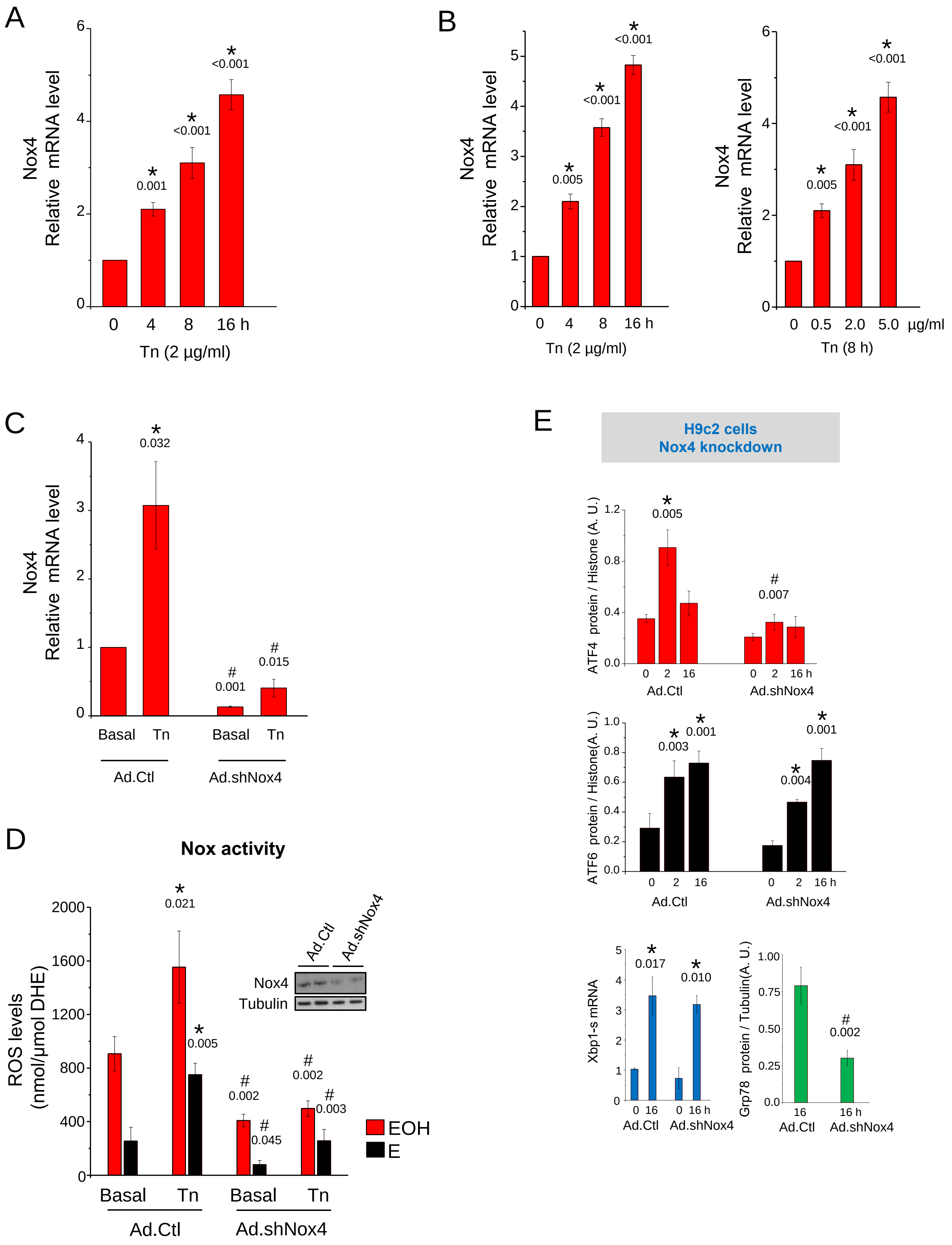
A
Nox4
Relative mRNA level
* 0.001
* <0.001
* <0.001
0 4 8 16 h
Tn (2 µg/ml)
B
Nox4
Relative mRNA level
* 0.005
* <0.001
* <0.001
0 4 8 16 h
Tn (2 µg/ml)
* 0.005
* <0.001
* <0.001
0 0.5 2.0 5.0 µg/ml
Tn (8 h)
C
Nox4
Relative mRNA level
* 0.032
# 0.001
# 0.015
Basal Tn Basal Tn
Ad.Ctl
Ad.shNox4
D
Nox activity
ROS levels
(nmol/µmol DHE)
* 0.021
* 0.005
# 0.002
# 0.045
# 0.002
# 0.003
Ad.Ctl
Ad.shNox4
Nox4
Tubulin
EOH
E
Basal Tn Basal Tn
Ad.Ctl
Ad.shNox4
E
H9c2 cells
Nox4 knockdown
ATF4 protein / Histone (A. U.)
* 0.005
# 0.007
0 2 16
0 2 16 h
Ad.Ctl
Ad.shNox4
ATF6 protein / Histone(A. U.)
* 0.003
* 0.001
* 0.004
* 0.001
0 2 16
0 2 16 h
Ad.Ctl
Ad.shNox4
Xbp1-s mRNA
* 0.017
* 0.010
0 16
0 16 h
Ad.Ctl
Ad.shNox4
Grp78 protein / Tubulin(A. U.)
0.002
16
16 h
Ad.Ctl
Ad.shNox4

Fig S1

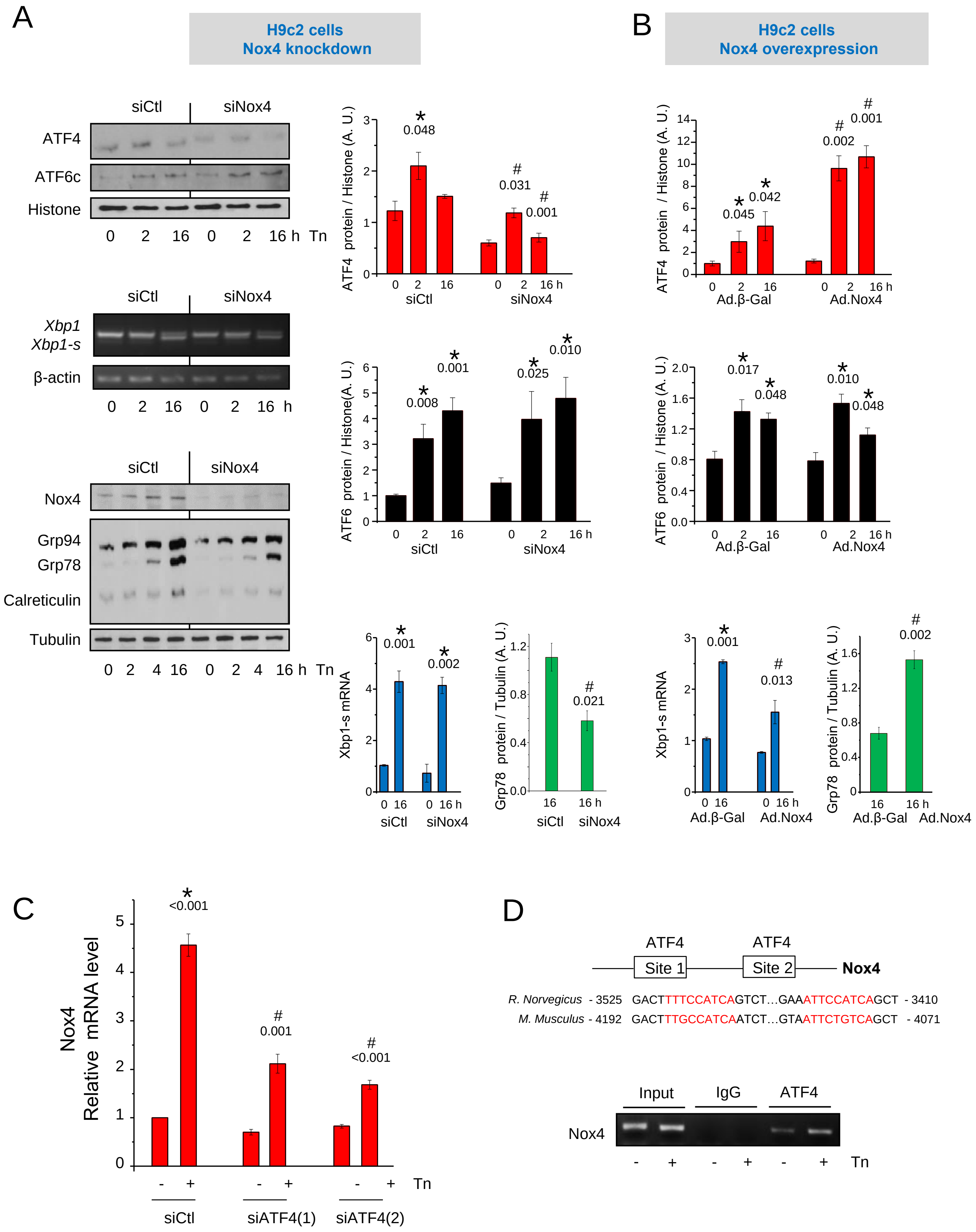
A
H9c2 cells
Nox4 knockdown
B
H9c2 cells
Nox4 overexpression
siCtl
siNox4
ATF4
ATF6c
Histone
0 2 16 0 2 16 h Tn
Xbp1
Xbp1-s
β-actin
Nox4
Grp94
Grp78
Calreticulin
Tubulin
0 2 4 16 0 2 4 16 h Tn
ATF4 protein / Histone (A. U.)
ATF6 protein / Histone(A. U.)
Xbp1-s mRNA
Grp78 protein / Tubulin (A. U.)
Ad.β-Gal
Ad.Nox4
C
Nox4 Relative mRNA level
siATF4(1)
siATF4(2)
D
ATF4
Site 1
Site 2
R. Norvegicus - 3525 GACTTTTCCATCAGTCT…GAAATTCCATCAGCT - 3410
M. Musculus - 4192 GACTTTGCCATCAATCT…GTAATTCTGTCAGCT - 4071
Input
IgG

Fig S2

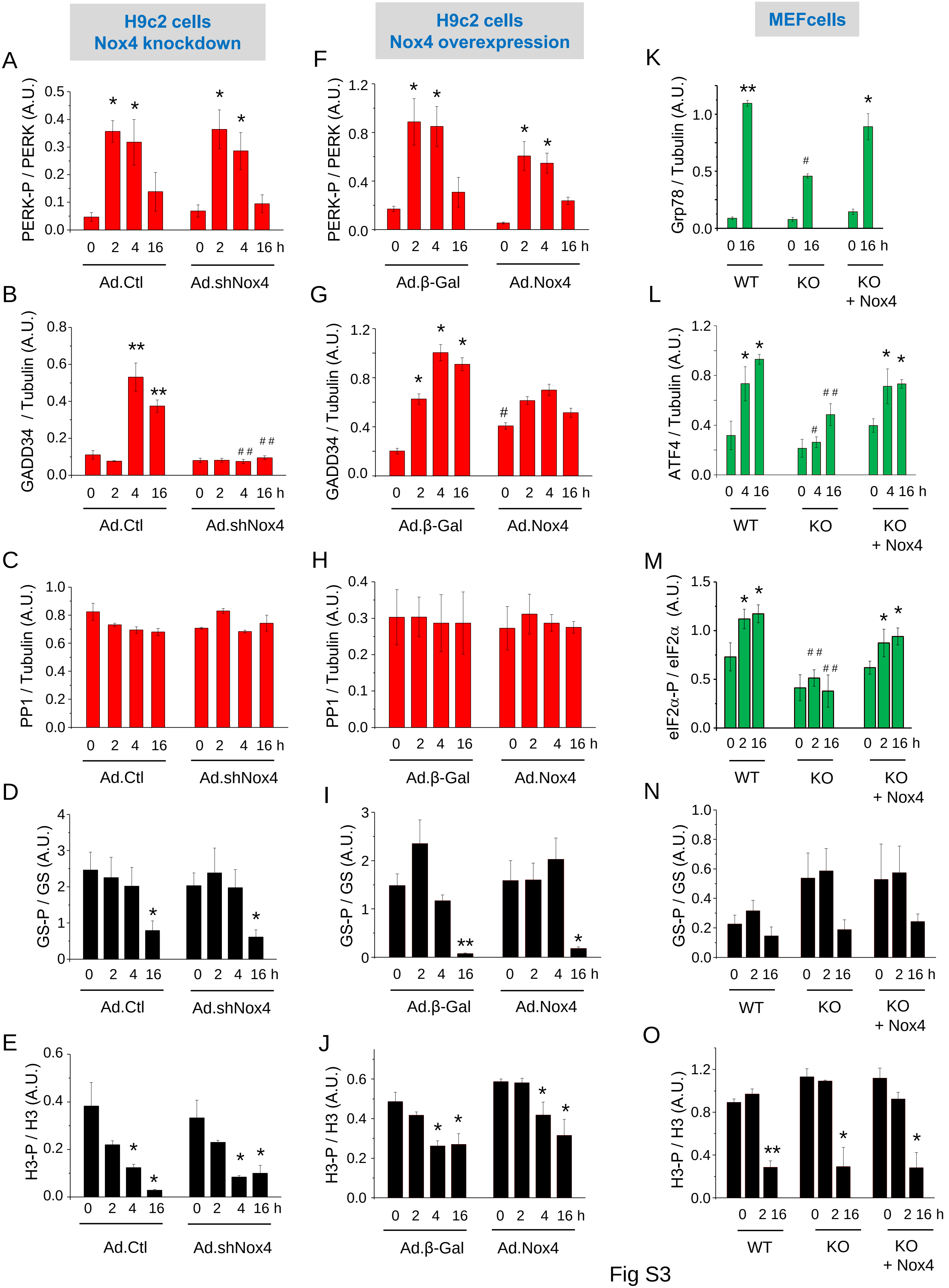
H9c2 cells
Nox4 knockdown
H9c2 cells
Nox4 overexpression
MEFcells
A
PERK-P / PERK (A.U.)
0 2 4 16 0 2 4 16 h
Ad.Ctl
Ad.shNox4
B
GADD34 / Tubulin (A.U.)
C
PP1 / Tubulin (A.U.)
D
GS-P / GS (A.U.)
E
H3-P / H3 (A.U.)
F
G
H
I
J
Ad.β-Gal
Ad.Nox4
K
Grp78 / Tubulin (A.U.)
0 16 0 16 0 16 h
WT
KO
KO
+ Nox4
L
ATF4 / Tubulin (A.U.)
0 4 16 0 4 16 0 4 16 h
M
eIF2α-P / eIF2α (A.U.)
0 2 16 0 2 16 0 2 16 h
N
O

Fig S3

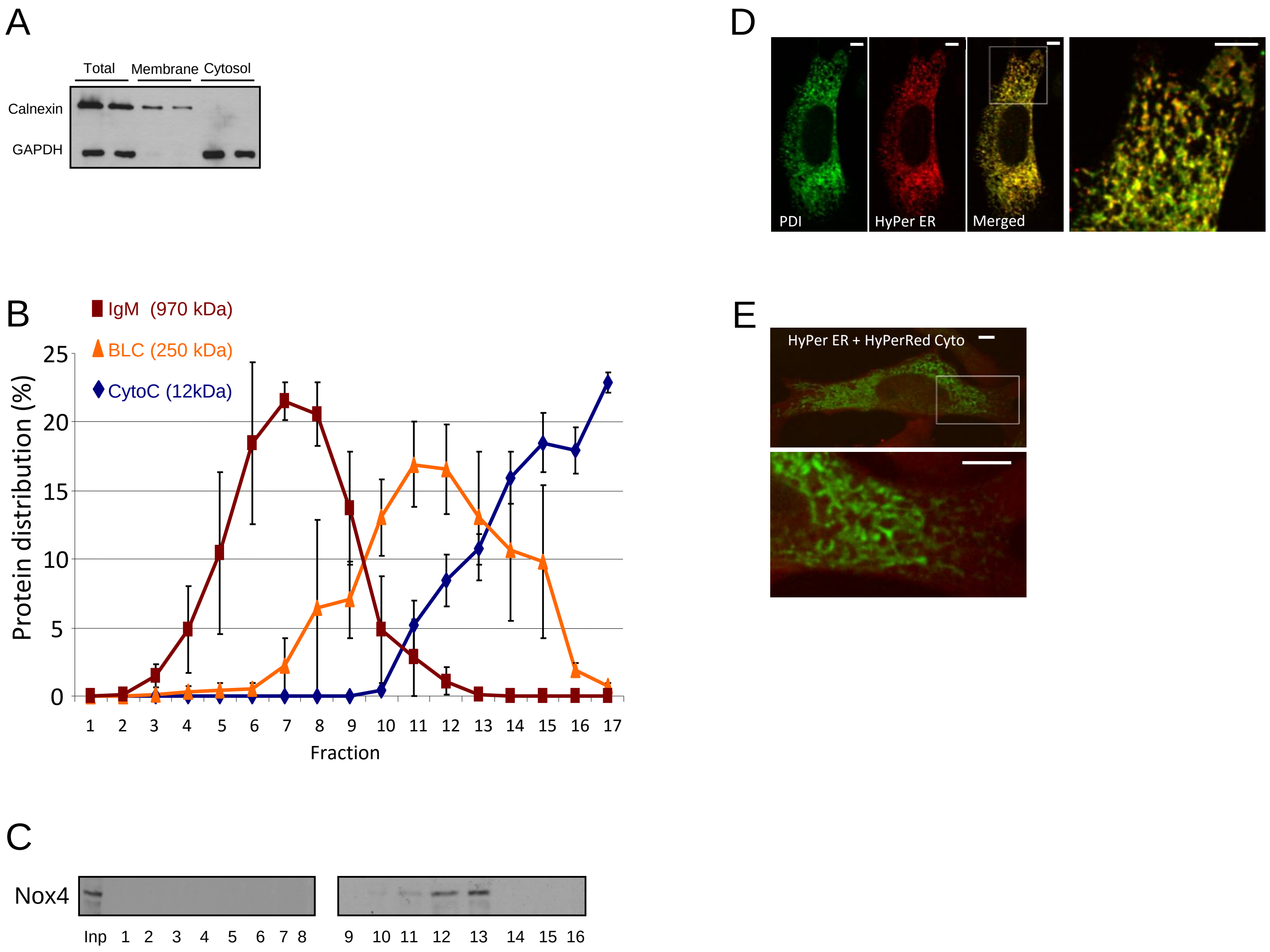

A
Total
Membrane
Cytosol
Calnexin
GAPDH
B
IgM (970 kDa)
BLC (250 kDa)
CytoC (12kDa)
Protein distribution (%)
0
5
10
15
20
25
1 2 3 4 5 6 7 8 9 10 11 12 13 14 15 16 17
Fraction
C
Nox4
Inp 1 2 3 4 5 6 7 8
9 10 11 12 13 14 15 16
D
PDI
HyPer ER
Merged
E
HyPer ER + HyPerRed Cyto

Fig S4

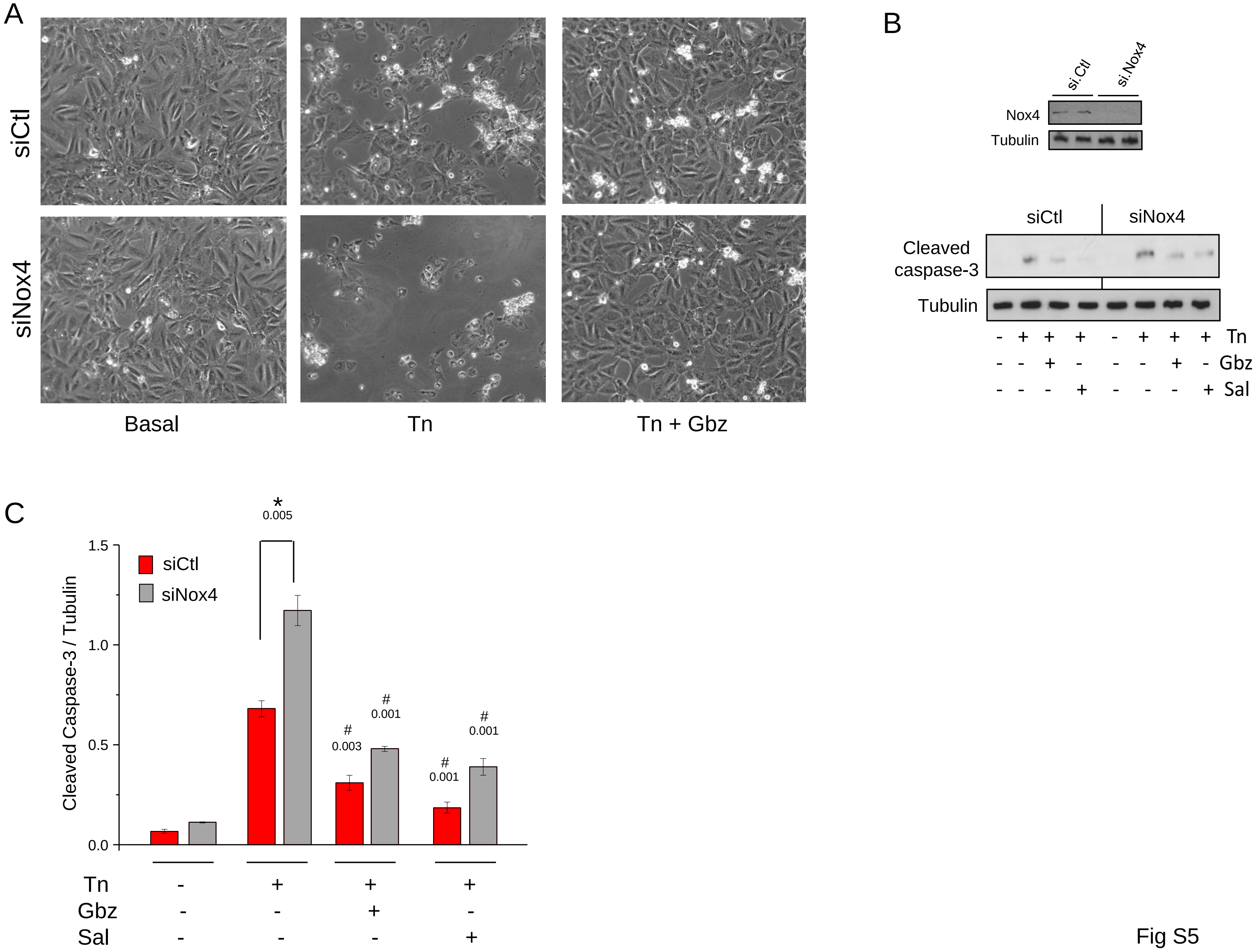
A
siCtl
siNox4
Basal
Tn
Tn + Gbz
B
si.Ctl
si.Nox4
Nox4
Tubulin
siCtl
siNox4
Cleaved caspase-3
Tubulin
- + + + - + + + Tn
- - + - - - + - Gbz
- - - + - - - + Sal
C
Cleaved Caspase-3 / Tubulin
1.5
1.0
0.5
0.0
siCtl
siNox4
*
0.005
#
0.003
#
0.001
#
0.001
#
0.001
Tn - + + +
Gbz - - + -
Sal - - - +

Fig S5
