# Supplementary material for: Targeted redox inhibition of protein phosphatase 1 by Nox4 regulates eIF2α‐mediated stress signaling
Source: EMBO J. 2016 Jan 7;35(3):319–34. doi: 10.15252/embj.201592394 (PMC4741303; doi:10.15252/embj.201592394)
Supplement: Supplementary file 2 — Expanded View Figures PDF [file EMBJ-35-319-s002.pdf]

Expanded View Figures

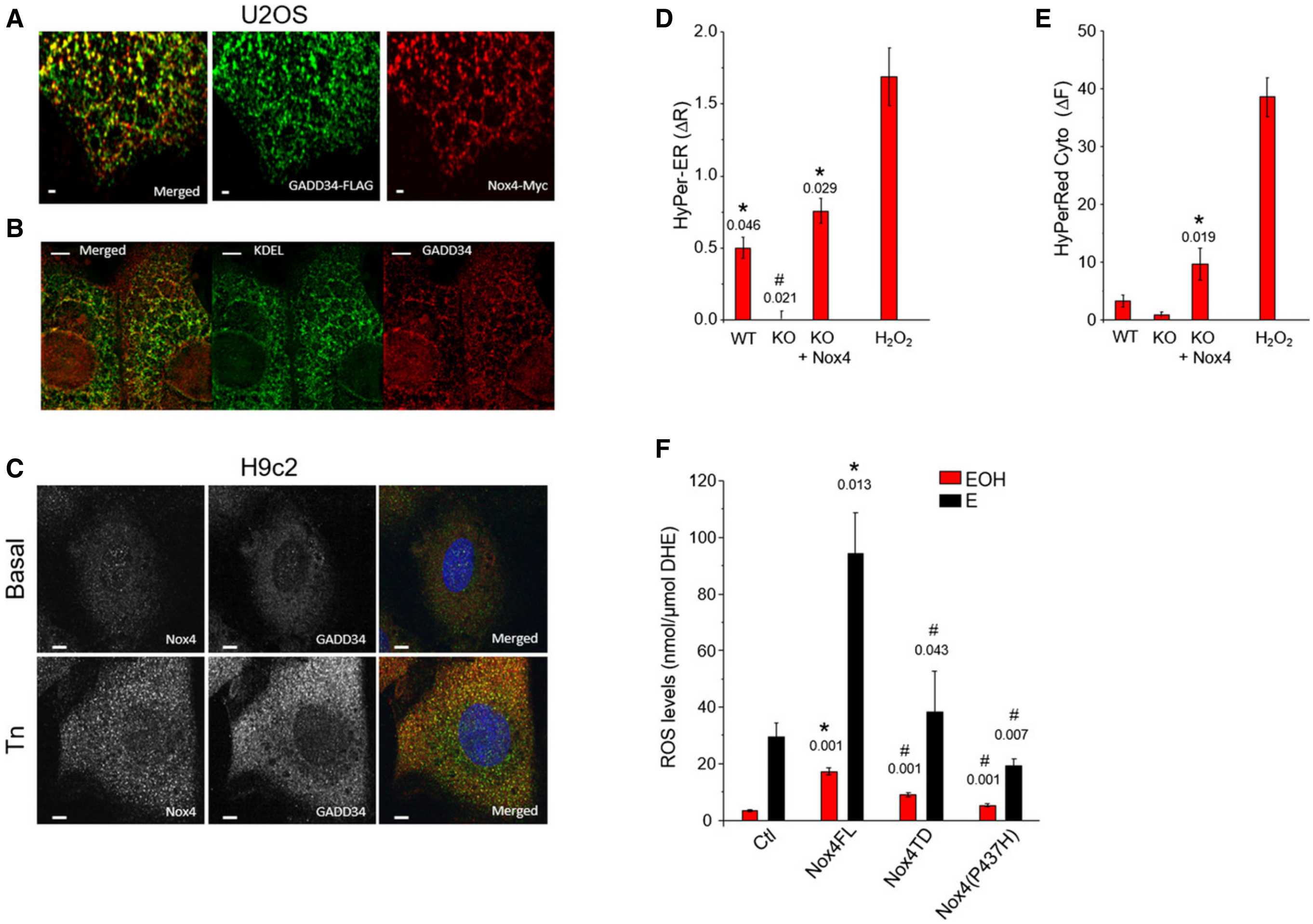

Figure EV1. Association of Nox4 with GADD34 at the ER.

A Confocal microscopic images of U2OS cells co-transfected with Myc-tagged Nox4 and Flag-tagged GADD34 showed a co-localization of Nox4 (red) and GADD34 (green). Yellow dots in the top panel denote co-localization of fluorescence signals. Scale bars, 1 µm.

B Confocal microscopic images of U2OS cells co-transfected with Myc-tagged Nox4 and Flag-tagged GADD34 as in (A), then stained for GADD34 (red) and KDEL (green) as an ER marker. GADD34 co-localized with KDEL. Scale bars, 5 µm.

C Confocal microscopic images of H9c2 cells treated with tunicamycin (Tn, 2 µg/ml, 6 h), showing increased endogenous levels and co-localization between Nox4 (green) and GADD34 (red). Scale bars, 10 µm.

D, E Mean ± SEM data for changes in HyPer-ER and HyPer-Red Cyto fluorescence from 3 independent cell preparations/group and at least 12 cells imaged/preparation. *, significant comparing Tn versus basal; #, significant comparing KO versus other groups. Extracellular H_2O_2 (200 nM) was added as a positive control, and the changes in HyPer-ER and HyPer-Red Cyto fluorescence were quantified.

F ROS levels were increased in HEK293 cells transfected with full-length Nox4 (Nox4FL), but not with Nox4 transmembrane domain (Nox4 TD) or a Nox4(P437H) mutant. ROS levels were measured by HPLC-based quantification of the dihydroethidium (DHE) oxidation products, 2-hydroxyethidium (EOH) and ethidium (E). n = 4/group. *, significant comparing full-length Nox4 versus control (Ctl); #, significant comparing full-length Nox4 versus Nox4 TD or Nox4 P437H.

Data information: Data are presented as mean ± SEM. Comparisons were made by Student's t-test or one-way ANOVA, with $P < 0.05$ considered significant. Values above bar graphs denote the level of significance.

© 2016 The Authors

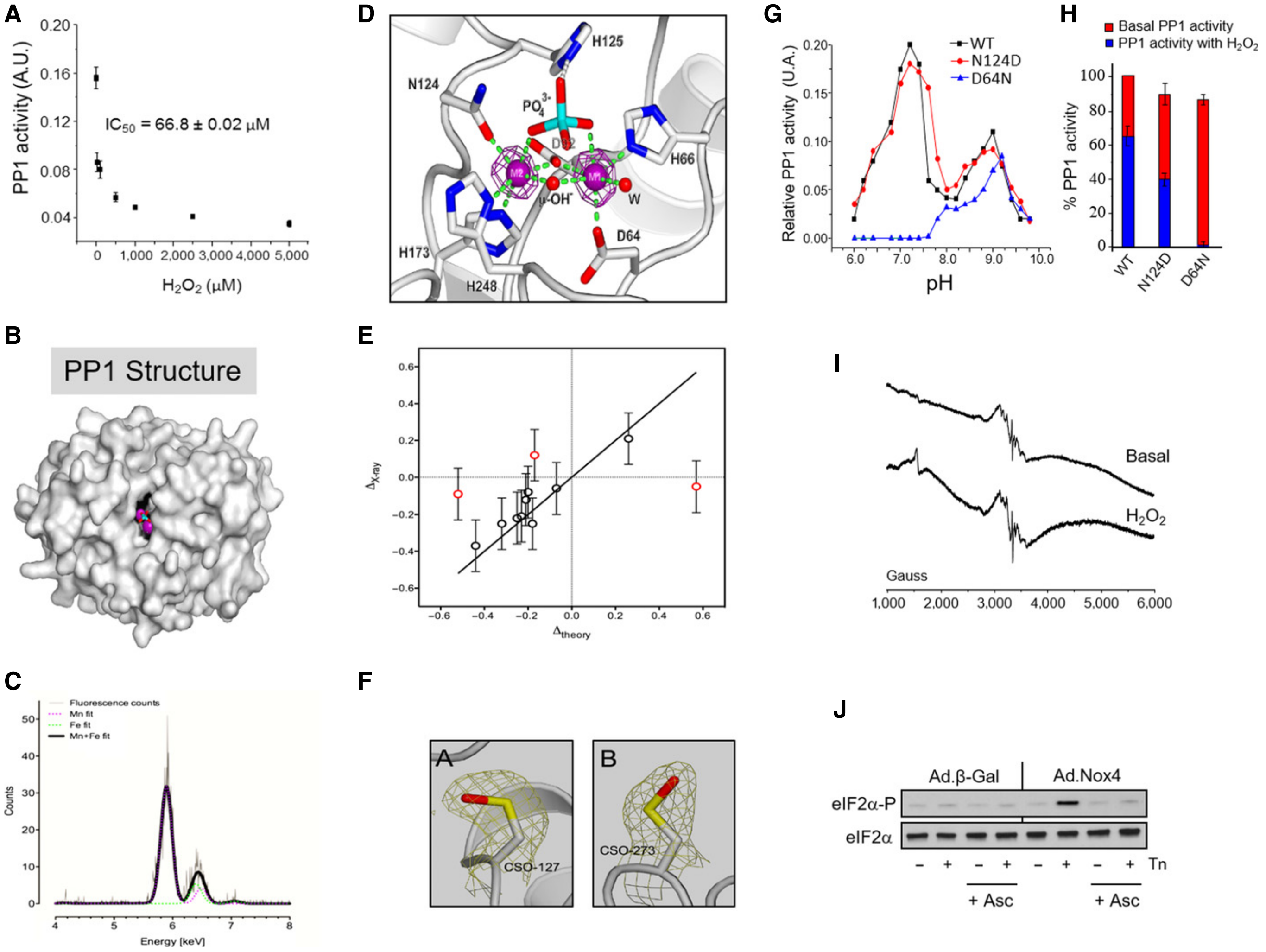

Figure EV2.

 © 2016 The Authors

Figure EV2. X-ray crystallographic features of the PP1 metal coordination center and its redox regulation.

A Purified recombinant PP1 was dose-dependently inhibited by H_2O_2.

B Overall representation of the PP1 catalytic domain. The catalytic Mn-Mn or Mn-Fe metal ions shown as magenta spheres are located in a shallow groove at the molecular surface. PP1 side chains coordinating the metal ions and forming the base of the groove are highlighted in black. A phosphate ion, shown in stick representation with phosphate and oxygen atoms, colored cyan and red, respectively, is bound to the dinuclear center.

C The X-ray fluorescence emission spectrum of PP1 crystals reveals a mixture of Mn and Fe ions with the latter ion typically present in a lower amount. The ratio for the sample shown here is Mn:Fe 1:0.175. No other metals are present at significant concentration. The emission spectrum in the 4–8 keV energy region is shown by the gray thin line. Fitting for the individual Mn (purple dotted line, expected emission energies 5,899 eV and 6,491 eV) and Fe (green dotted line, expected emission energies 6,404 eV and 7,058 eV) metals as well as the total fit (black continuous line) are also shown. Fitting was carried out with the package PyMca (Solé *et al*, 2007). Excitation energy was 18 keV.

D Cartoon representation of the PP1 active site with the dinuclear (M1, M2) metal center represented by purple spheres. The metal ions are pseudo-octahedrally coordinated. Residues at coordinating distance (D64, H66, D92, N124, H173, H248) as well as the catalytically important H125 hydrogen-bonded to the phosphate moiety are shown as sticks. P, N, C, O atoms are in cyan, blue, white, and red, respectively. The bridging μ-OH^- and the terminal water (W) are represented by small spheres. In crystallo metal analysis shows the presence of both Mn and Fe metals with the former being the most abundant. Anomalous difference maps (shown in purple at the +8σ level) calculated from data collected at the 6,876.6 eV energy where the Fe anomalous contribution is negligible indicate that Mn is present at both M1 and M2 centers. An attempt to specifically locate Fe ions using a double difference anomalous map approach (Than *et al*, 2005) was unsuccessful likely owing to the low Fe content.

E Correlation between change in metal–ligand (M–L) coordination distances determined by X-ray experimental and theoretical methods. There is a good correlation between theory and experiment with 75% of the Δ values (black circles) lying on the diagonal within error while three values (red circles) can be considered outliers. Most points lie on the lower-left quadrant, implying a contraction of the average (M–L) distance upon metal oxidation. See Appendix Table S1 and related text for further details.

F PP1 cysteine residues Cys127 (A) and Cys273 (B) were often seen oxidized to their sulfenic acid (CSO) derivative. Occasionally, Cys291 is also oxidized. 2mFo-DFc electron density maps are shown in blue at the 1.1σ level. S, C, O atoms are in yellow, white, and red, respectively. Cys oxidation appears independently of H_2O_2 treatment as we have observed sulfenic acid derivatization also in ascorbate-treated crystals. Cysteine oxidation does not affect PP1 catalysis as the PP1 C127, 273S double variant displays the same activity as WT PP1 (see Fig 4A).

G, H Effect of H_2O_2 on the activity of WT PP1 and PP1 variants bearing mutations in amino acids involved in metal coordination, N124D and D64N. The pH optimum for each PP1 variant was determined by assessing PP1 activity over a range of pH values (buffers: 100 mM Tris–HCl pH 6–8 and 100 mM glycine–NaOH pH 9–10) (G). The pKa for WT PP1 and N124D PP1 was 7.2, and for D64N PP1, it was 9.8. The PP1 variants were incubated with 0.1 mM H_2O_2 for 20 min at 37°C, and PP1 activity was assessed at optimum pH (H). The red bars show PP1 activity under reduced conditions and the blue bars after H_2O_2 treatment. The relative inhibition by H_2O_2 was substantially increased in the mutant proteins.

I Low-temperature EPR spectra of purified PP1 at baseline and after treatment with H_2O_2 (1 mM). The central six-line signal from 2.5 to 4.5 kG is consistent with Mn^{2+}. After H_2O_2 treatment, there is a consistent signal in the region of 1.5 to 2 kG, which is typical for Fe(III).

J Effect of ascorbate (Asc, 0.5 mM) on eIF2α phosphorylation in tunicamycin (Tn)-treated H9c2 cells with overexpression of Nox4 (Ad.Nox4). The Nox4-induced increase in eIF2α phosphorylation after 4 h of Tn was inhibited by ascorbate (see also Fig 4F).

Data information: Data are means ± SEM or representative of at least three independent experiments.

© 2016 The Authors
